# Performance of the Hendrich Fall Risk Model II in Patients Discharged from Rehabilitation Wards. A Preliminary Study of Predictive Ability

**DOI:** 10.3390/ijerph18041444

**Published:** 2021-02-04

**Authors:** Isabella Campanini, Annalisa Bargellini, Stefano Mastrangelo, Francesco Lombardi, Stefano Tolomelli, Mirco Lusuardi, Andrea Merlo

**Affiliations:** 1 LAM-Motion Analysis Laboratory, Neuromotor and Rehabilitation Department, S. Sebastiano Hospital, Azienda USL-IRCCS di Reggio Emilia, Via Circondaria 29, 42122 Correggio, Italy; andrea.merlo@ausl.re.it; 2 Department of Biomedical, Metabolic and Neural Sciences, University of Modena and Reggio Emilia, Via Giuseppe Campi, 287, 41125 Modena, Italy; Annalisa.Bargellini@unimore.it; 3 Clinical Governance Unit, Azienda USL-IRCCS di Reggio Emilia, Via Giovanni Amendola, 2, 42122 Reggio Emilia, Italy; Stefano.Mastrangelo@ausl.re.it; 4 Neurorehabilitation Unit, Neuromotor and Rehabilitation Department, S. Sebastiano Hospital, Azienda USL-IRCCS di Reggio Emilia, Via Circondaria 29, 42015 Correggio, Italy; Francesco.Lombardi@ausl.re.it; 5 Motor Rehabilitation Unit, Neuromotor and Rehabilitation Department, S. Sebastiano Hospital, Azienda USL-IRCCS di Reggio Emilia, Via Circondaria 29, 42015 Correggio, Italy; Stefano.Tolomelli@ausl.re.it; 6 Neuromotor and Rehabilitation Department, Azienda USL-IRCCS di Reggio Emilia, Via Circondaria 29, 42015 Correggio, Italy; Mirco.Lusuardi@ausl.re.it

**Keywords:** falls risk assessment, Hendrich Fall Risk Model II, rehabilitation units, discharge to home

## Abstract

(1) Background: Falls are a dangerous adverse event in patients discharged from rehabilitation units, with the risk of falling being higher in the first weeks after discharge. In this study, we assessed the predictive performance of the Hendrich Fall Risk Model II tool (HIIFRM) when administered before discharging patients to their home from rehabilitative units in orthopedic (OR), neurologic (NR) and pulmonary (PR) rehabilitation wards. (2) Methods: Over a 6-month period, all adult patients who returned home after discharge were assessed by HIIFRM. At six months from discharge the occurrence of falls was obtained by performing a structured survey. The HIIFRM predictive performance was determined by the area under the ROC curve (AUC), sensitivity (Se) and specificity (Sp) for the whole sample and split by ward. (3) Results: 85 of 141 discharged patients were living at home and agreed to take part in the survey. Of these, 19 subjects fell, 6 suffered fractures or head traumas and 5 were hospitalized. The AUC was 0.809 (95% CI: 0.656–0.963), Se was 0.67 (0.30–0.93) and Sp was 0.79 (0.63–0.90) for OR patients. (4) Conclusions: Our preliminary results support the use of HIIFRM as a tool to be administered to OR patients at discharge and provides data for the design of a large study of predictive ability.

## 1. Introduction

Falls are the most common adverse event occurring in care facilities and hospitals. Over 80% of all adverse events in hospitals are related to falls, with the fall rate being strongly dependent on the case mix and the settings [1]. The two settings where falls are more frequent are geriatric units and rehabilitation wards. Here the fall rate can be as high as 20 falls per 1000 patient bed days [2,3,4,5,6,7].

When dealing with falls, the post-discharge period can be critical [8]. This is particularly true for those patients with a very high risk of falling, such as the elderly and rehabilitative patients. Up to 40% of older adults discharged from hospital fall at least once during the 6-month period following discharge and more than 50% of these falls result in a serious injury [8,9]. When focusing on neurological patients, fall rates ranging between 46% and 73% have been reported for the first six months after discharge [10,11,12].

Community-dwelling older adults are at risk of falling too. Epidemiologic data indicate that one elderly person out of three experiences a fall at least once a year. This incidence increases to 1:2 for persons over the age of 80 and nearly doubles in subjects living in nursing homes [13].

During hospitalization, the risk of patients falling can be categorized from low to high by means of custom designed tools that monitor the presence of known risk factors and assign them a score based on previously determined odd ratios. The systematic review and meta-analysis by Park, which analyzed 33 prospective studies for a total of 9743 subjects, provided scientific evidence for a selection of setting-dependent fall risk assessment tools to properly predict the occurrence of falls, both for hospitals and community living. This review provides a comprehensive analysis of the pooled sensitivity and specificity of the available fall risk tools, along with their inter-study variability [14]. Those with the best performances showed pooled sensitivities and specificities ≥70% and low interstudy variability [14].

Balance assessment tools validated for the community-dwelling elderly, such as the Berg Balance Scale (BBS) and the Timed Up and Go (TUG) tests, typically address the balancing ability in everyday activities [14] and also when standing upright [15]. As many as 24 different tools have been described in the literature [14]. Among these, BBS presented a pooled sensitivity and specificity of >0.7, but with large heterogeneity among studies. The TUG test presented a pooled sensitivity of approximately 0.7 despite a low pooled specificity (0.5). Similar results were found for the Tinetti Balance scale [14].

Fall risk tools validated for inpatients, such as the Hendrich Fall Risk Model II (HIIRFM) and the St. Thomas’s Risk Assessment Tool in Falling Elderly Inpatients (STRATIFY), address physiological items such as consciousness, urinary function and drug intake, and may include one or more items related to patients’ physical abilities, in a multifactorial approach [16,17]. When focusing on rehabilitative units, the Stroke Assessment Fall Risk, the 4-Item Falls Assessment Tool Scale and the HIIRFM have also been addressed in the literature [18,19,20]. These showed moderate to good predictive abilities, with sensitivity and specificity in the order of 70–80%, and low inter-study variability for sensitivity but high inter-study variability for specificity [Park]. Of note, falls are frequent in young adults with neurological conditions, too [20].

In a recent study by Hendrich and colleagues on more than 200,000 patients, HIIFRM showed sensitivity and specificity of 79% and 64%, respectively [21].

While the predictive ability of fall risk screening tools has been addressed for both inpatients and outpatients, very little information is available on the usability of these tools at discharge with regards to identifying subjects at high risk of falling after their home discharge. In this study, we assessed the HIIFRM score in a sample of patients at their discharge from a rehabilitative hospital including the neurologic, orthopedic and pulmonary rehabilitation units. Next, we collected their fall history using a structured survey at six months from discharge. The prospective predictive performance of HIIFRM delivered at discharge was assessed in terms of sensitivity and specificity for orthopedic, pulmonary and neurologic rehabilitative patients.

## 2. Materials and Methods

### 2.1. Study Design and Setting

This was a 1-year-long prospective observational study. A 6-month enrollment phase was followed by a 6-month follow-up period. All adult patients (age ≥ 18 years) discharged from the Orthopedic Rehabilitation (OR), Pulmonary Rehabilitation (PR), or Neurological Rehabilitation (NR) Units of the Correggio Hospital (AUSL-IRCCS di Reggio Emilia) during the study period were considered for enrolment. NR typically receives patients from the stroke unit whilst OR typically receives patients from the orthopedic units of our institution, who had undergone prior orthopedic surgery.

### 2.2. Patients

All adult patients discharged to their homes were included in this study. Patients were excluded only when moved to other wards, residential facilities or in case of further hospitalization. No other inclusion/exclusion criteria were used.

All patients gave an informed signed consent to data treatment and agreed to be contacted at six months after discharge. The study was approved by the Ethical Committee of Reggio Emilia, protocol number 2017/0123702.

### 2.3. Fall Risk Assessment Procedure at Discharge

At discharge, patients underwent a fall risk assessment by HIIFRM [16]. This tool consists of 8 weighted items: confusion/disorientation/impulsivity (score = 4), symptomatic depression (score = 2), altered elimination (score = 1), dizziness or vertigo (score = 1), male sex (score = 1), antiepileptic prescription (score = 2), benzodiazepine prescription (score = 1) and ‘‘get up from chair’’ test (score ranging between 0 and 4). The total score of HIIFRM can range between 0 and 16 (where 16 is the highest risk). Patients with a score ≥5 are classified at risk of falling [16]. When patients are not able to carry out the “get up from chair” test, they were classified at risk if the total score of the remaining items was equal to or higher than the threshold of 5 [16]. All patients were assessed by two trained physiotherapists (PTs), according to the instructions provided in [16,22]. It is worth pointing out that, in this study, we tested the tool performance outside of its operational definitions, which refer to elderly inpatients only.

We used the scale as published in 2003 [16]. In 2005, a comprehensive method and system for fall risk assessment referred to as Hendrich II Fall Risk Model^®^ was patented in the US (US 2005/0182305) and its use is subject to licensing (see https://hendrichfallriskmodel.com).

### 2.4. Survey on Post-Discharge Falls

At six months from discharge, patients received a detailed standardized interview on their fall history. A single trained interviewer contacted each subject (or caregiver). The survey was intended to record falls, the dynamic of each fall and its consequences on both the subject’s health and the additional burden to the health care system; the occurrence of soft tissue injury, fractures and head traumas was collected, along with the individual’s access to their general practitioner, the emergency service and the need for further surgery and/or hospitalization consequent to the fall. According to the literature, a fall was recorded when “an event which results in a person coming to rest inadvertently on the ground or floor or other lower level took place” [23].

### 2.5. Statistical Analysis

Descriptive statistics were used to characterize the sample as a whole and also for each individual ward. The group difference in the HIIFRM score between fallen and not fallen subjects was investigated with the non-parametric Mann–Whitney test. Statistical significance was set at 5%. The predictive power of HIIFRM was obtained by computing the area under the ROC curve (AUC), along with its 95% confidence interval (95% CI). A contingency table merging fall predictions and fall occurrences was filled in by entering the number of subjects classified at risk (yes/no) in the rows and the number of subjects fallen during the 6 months after discharge (yes/no) in the columns. Sensitivity (Se), specificity (Sp) and positive and negative predicted values (PPV, NPV) were computed along with their 95% CI.

### 2.6. Sample Size Computation for Future Studies

Finally, based on the experimental values obtained in this preliminary study, the number of subjects needed to conduct a comprehensive study of predictive ability was computed as follows (Equation (1)):

(1) $$N=\;\frac{{\left(z_{1-\alpha /2}\right)}^{2}}{W^{2}}\;\cdot \mathrm{Se}\cdot \left(1-\mathrm{Se}\right)\cdot \frac{1}{\mathrm{Prev}}\cdot \frac{1}{\left(1-\mathrm{DOR}\right)}$$

where *z*_1−*α*/2_ = 1.96, *W* is the desired half-width of the 95% confidence interval of Se, Prev is the prevalence of falls and DOR is the observed drop-out rate [24].

## 3. Results

### 3.1. Sample Characteristics

**Figure 1:**
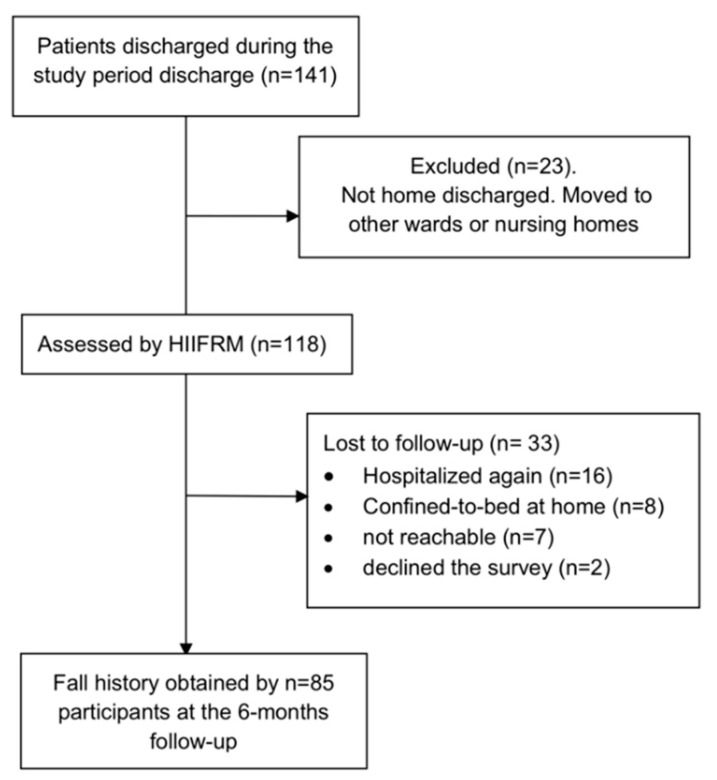
Flow diagram of patients assessed in the study from the date of discharge to the follow-up at the 6-month mark.

A total of 141 consecutive adult patients were discharged from the rehabilitation wards during the enrollment period. Of these, 23 were moved to other wards or residential facilities. The remaining 118 patients were included in the study. Figure 1 presents the flow diagram showing the number of participants evaluated and the reasons for dropouts, from the initial enrollment to the time of the survey.

In our sample, the main condition that had led to admission to NR was stroke, followed by traumatic brain injury, specific rehabilitation following functional surgery and other neurological conditions. Nearly half of NR patients were using aids or orthoses at discharge. Two of them had cognitive problems. The OR ward received patients from the orthopedic surgery unit. These were mainly patients who had undergone total hip replacement, total knee replacement or patients with polytraumas. Many of them presented age-related comorbidities and two patients had a concomitant neurological disease (e.g., stroke or parkinsonism). Finally, patients were admitted to PR because of severe respiratory failure, chronic obstructive pulmonary disease or in order to manage their adaptation to continuous positive airway pressure therapy in severe obstructive sleep apneas.

These patients are part of the patients who participated in our previous study on the predictive ability of the HIIFRM tool, when administered upon admission to rehabilitative wards [20].

At the 6-month mark, 85 patients were community-dwelling people, and agreed to be interviewed. Of the remaining 33 subjects, 16 had been hospitalized again, 8 were confined to bed at home, 7 were not reachable (people from abroad returning home or the telephone number was non-existent), and for 2 their caregiver turned down the call. The vast majority of dropouts were neurological patients.

Sample average characteristics are reported in Table 1. Values are presented both for the sample as a whole, and then by the rehabilitation discharge ward of origin. While a few young adults were present, four out of five patients in the whole sample had an age of 60 years or more and four out of five patients in the OR group had an age of 67 years or more.

### 3.2. Fall Risk Classification at Discharge

Median HIIFRM score at discharge is reported in Table 1. Based on the HIIFRM classification procedure (score ≥ 5), 35 (41%) patients were classified at high risk of falling after discharge. Furthermore, approximately 3 out of 4 patients from NR were considered at risk of falling at the time of discharge. This ratio dropped to approximately 1 out of 3 for patients coming from OR and to 1 out of 7 at PR.

The percentage occurrence of each single item of the HIIFRM is reported in Table 2. NR patient scores were at or above the cut-off threshold of 5 mainly because of the item confusion/disorientation/impulsivity, which alone scores 4. OR and PR patients reached the threshold score due to a combination of items.

### 3.3. Fall History and Characteristics of Fall Dynamics

Out of the 85 interviewed subjects, 19 fell (age range 20–91 years) during the 6 months following their discharge, resulting in a 22% fall rate. As summarized in Table 3, falls occurred for 9 patients out of 26 from NR (9/26 = 35%), 9 patients out of 46 from OR (9/46 = 20%), and 1 patient out of 13 (1/13 = 8%) from PR.

No significant age difference was found between fallers and non-fallers (Mann–Whitney test, *p =* 0.33). The fallen PR patient was 64 years old. Among NR fallers, age ranged between 20 and 75 years, and was lower than that of OR patients, where age ranged between 41 and 91 years (Mann–Whitney test, *p* < 0.001). This was due to the presence of young individuals that were admitted to the NR ward because of traumatic brain injuries.

A complete description of fall dynamics for each subject is reported in Table 4, along with the actual cause of the fall. A total of 8 out of 19 fallen subjects needed access to emergency services. Of these, 5 suffered a fracture, i.e., 1 fracture for every 4 falls. When comparing the whole sample, the rate of fractures was 5.8% (5/86). Five subjects were admitted to hospital and three subjects needed surgery. In the remaining cases, the fallen subjects were referred back to their general practitioner.

### 3.4. Between Groups Comparison

**Figure 2:**
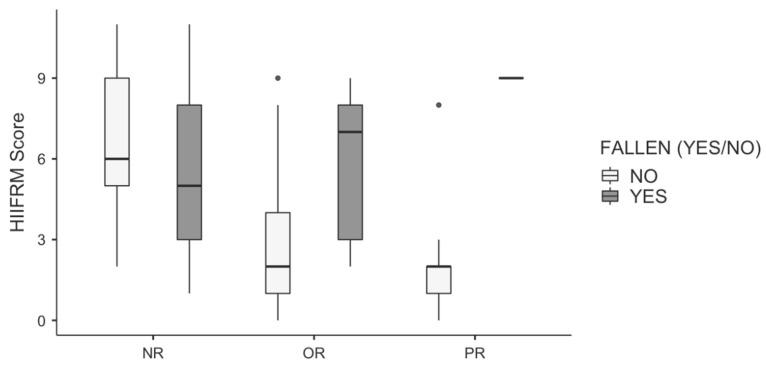
Boxplot of the HIIFRM score for fallen and non-fallen patients, split by discharge ward. NR = neurological rehabilitation; OR = orthopedic rehabilitation; and PR = pulmonary rehabilitation.

The HIIFRM median score of all fallen subjects was 6 and greater than the value of not fallen subjects, which was equal to 3 for the whole sample (Mann–Whitney test, *p* = 0.005). The between-groups comparison of the HIIFRM score, split up into each individual discharge ward, is presented in Figure 2. The median score of OR fallen subjects was equal to 7 and significantly greater (*p* = 0.004) than that of not-fallen ones, the latter being 2, and with a minimum overlap between the distributions of these two groups. The single fallen PR subject had a score of 9, while PR non-fallen subjects had a median HIIFRM score of 2. Instead, the two box-plots of fallen and non-fallen NR patients were completely overlapping. It is evident from this figure that the HIIFRM had a good performance with orthopedic and pulmonary patients only.

### 3.5. Predictive Perfomrance of the HIIFRM When Administerd at Discharge

**Figure 3:**
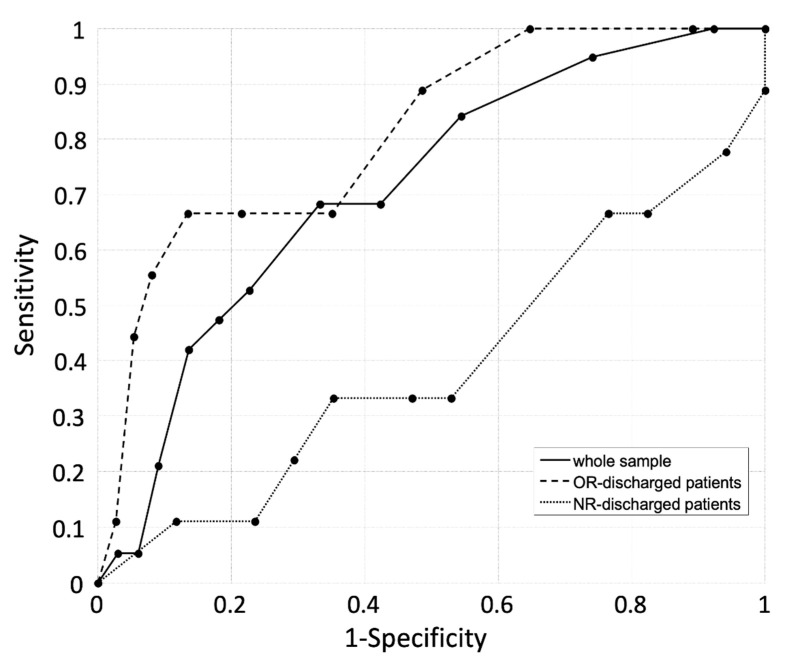
Receiver operating curve (ROC) computed on data collected from the whole sample (continuous line), from patients discharged from the orthopedic rehabilitation (OR) ward only (dashed line) and from patients discharged from the neurologic rehabilitation (NR) ward only (dotted line).

The HIIFRM predictive performance was calculated based on the 19 falls from the overall 85 subjects. The ROC curve obtained from data is presented in Figure 2. The area under the curve was AUC = 0.712, *p* = 0.005, (95% CI: 0.586–0.873), indicating a moderate predictive power, but with a very large confidence interval. Sensitivity was 68% (95% CI: 58–84%) and specificity was 66% (95% CI: 54–76%). When focusing on OR patients, the AUC rose to 0.809 (95% CI: 0.656–0.963), *p* = 0.004, indicating a very good predictive ability for this specific subsample. However, when focusing on the NR patients, the AUC was not significantly different from 0.5 (see Figure 3). Since only one PR subject fell, we did not compute the AUC.

The contingency tables between predicted and occurred falls are reported in Table 5, split by discharge wards, along with the values of Se, Sp, PPV and NPV. Interestingly, for OR-discharged patients, Se was 67% and Sp was as high as 79%. Out of 12 non-fallen PR subjects, 11 were correctly categorized (true negatives). This led to Sp being equal to 92% (95% CI: 76–100%). The single PR patient who fell was properly classified. However, we did not compute Se, as this would not have been reliable.

### 3.6. Sample Size Calculation for a Study of Predictive Ability

The sample size to be used in the design of a comprehensive study of predictive ability can be now obtained based on the values obtained in this preliminary study and by the formula reported in the Methods section.

When setting sensitivity to 67%, the desired half-width of the 95% confidence interval W at 10% and the prevalence of falls to 20%, the final number of people with the target condition (fallen) will be 86. If the expected prevalence of disease in the study sample is 20%, then the total number of participants included should be at least 430. This has to be incremented to take into account the expected dropout rate (DOR). In our study, DOR was lower than 10% for OR patients, resulting in a sample size of 478 subjects.

## 4. Discussion

This preliminary study of predictive ability was aimed at testing the predictive performance of HIIFRM in prospectively discriminating between future fallers and non-fallers, when administered at discharge and also obtaining preliminary estimates to properly design the sample size for a comprehensive study.

A moderate performance of the HIIFMR was found, with AUC = 0.712. This moderate performance resulted from a very good performance with the OR patients and a poor performance with the NR patients (see Table 5). Results suggest that HIIFRM looks promising for orthopedic patients (Se = 67%, Sp = 79%). The distribution in HIIFRM scores between OR future fallers and non-fallers was markedly different, as shown in Figure 2, thus explaining the very good [25] predictive performance of the tool (AUC = 0.809) for this subsample.

These results are perfectly in line with those reported by Hendrich and colleagues for inpatients [21]. In the literature, TUG and BBS have a pooled sensitivity of 0.7 when used with community-dwelling elderly [14]. This value is slightly better than the one we found, at the expense of a lower pooled specificity, varying between 0.5 and 0.7 [14]. It is worth noting that the idea of measuring the risk of falling at discharge with the aim of both planning preventive interventions and informing patients about their fall risk status is the novelty of this study. Since this is the first study in the literature addressing this topic, comparisons with the predictive capacity of other scales applied at discharge are not possible.

In our study, adequate specificity (92%) was found also for patients discharged from the PR ward. Unfortunately, sensitivity cannot be computed in this case since only one PR patient fell. On the other hand, HIIFRM at discharge was not suitable when used with neurologic patients, due to a lack in specificity (35%). In this case other tools focusing on the assessment of both functional and cognitive abilities are to be favored. Generally speaking, since NR patients are limited in number, when compared to OR patients, the delivery of at least a minimum set of preventive procedures to all NR patients seems to be advisable before discharge.

Due to the study’s characteristics, we collected information on the occurrence of falls during the six month period after discharge. Throughout the observation period, we were not able to establish precisely when these falls took place. Since in these high-risk subjects risk factors can change over time, fall risk assessment at discharge should be considered mainly as a tool to identify those subjects in need of immediate preventive interventions tailored to patients’ specific modifiable risk factors. Additionally, risk factors should be monitored over time via periodic assessments.

In our sample, OR-discharged patient falls occurred for different reasons, including fainting, walking, getting up from a chair and incontinence, as shown in Table 5. Many of these falls are related to either intrinsic or functional factors of fall risks that lead to predictable falls [26] and which can be addressed by a multifactorial tool. The HIIFRM total score combines the contribution of a set of intrinsic risk factors with the contribution of a functional test—the chair test—that assesses the residual force in the lower limbs (mainly quadriceps and glutei), their control and the overall balance during the transition. This item is key when evaluating OR patients discharged to their home, considering that it can score up to 4 when the threshold for being classified at risk is 5. Of note, the predictive ability of fall risk assessment tools based solely on the patient’s performance during the sit to stand transition has been described in the literature [27]. Falls due to OR patients tripping are generally related to the reduced ability to quickly compensate for disbalancing events and are linked with well-known age-related ailments including sarcopenia, increase in the sensory detection thresholds, decrease in nerve conduction velocity and central processing capacity [28]. It is worth pointing out that, according to Morse’s definition, 2 out of 3 false positive subjects (see Table 5) fell for unpredictable circumstances [26]. This situation is just as common in fall risk studies as it is in real life, and no fall risk assessment tool can predict them. One fall was caused by a fainting spell after introducing a new medication and the other subject fell whilst hopping on a bicycle.

Instead, in NR patients the most frequent cause of falling was tripping while attempting to walk briskly. We hypothesize that this phenomenon can explain the limited performance of the HIIFRM tool in identifying future fallers among NR-discharged patients. While this tool showed adequate predictive ability with acute NR inpatients [20], it might underestimate their functional (e.g., equinus foot deviation) and cognitive impairments after discharge, such as the tendency for acting out risky behavior in younger NR subjects. These aspects can be better assessed by other tools that evaluate balance, gait, functional independence and previous fall history [29]. For example, being classified at high risk by the Falls Risk for Older People in the Community (FROP-Com) led to an increased likelihood of falling by 4.5 times in the first 12 months after being discharged from the rehabilitation ward after a stroke [30].

Just 1 of the 13 patients discharged from the PR ward fell during the 6-month follow-up period. The median HIIFRM score of non-fallers was 2. This number is well below the cut-off score of 5. Hence, we hypothesize that this tool could be useful when assessing these patients, even though data from a wider sample are necessary to validate this assumption.

The minimum clinically important difference for HIIFRM is not described in the literature, since it is used as a classifier. Nonetheless, a recent study by Hendrich and colleagues on >200,000 inpatients showed an increase in the patients’ fall rate from 10% up to 60% when the HIIFRM score increased from 3 to 7, with a nearly linear increase of +12.5% in fall rate at each one point increment of the score [21]. This result can be useful when discussing the clinical meaning of the differences between scores in repeated assessments or when comparing groups, as in Figure 2.

It is conceivable that identifying subjects at high risk of falls before their discharge can be useful as a means of targeting preventive interventions on a limited number of subjects, thus increasing their feasibility and efficacy. The very low rate of false negatives obtained for OR patients (3/33 = 9%) further supports the use of the HIIFRM when the goal is delivering preventive efforts only to a limited and selected proportion of subjects, with well-identified risk factors and those at very high risk of falling within six months after discharge. Moreover, these risks can be shared with patients for educational purposes, to make them understand their impairment, especially the lifestyle changes to be made once they return home, including the acceptance of home modifications suggested by occupational therapists (OTs). These changes can reduce risky behavior and lessen potential hazards.

The overall fall rate found in our study in the six months after discharge was 22%. This number is much lower than that reported by Hill and colleagues, which was 40%. Their study monitored a cohort of 343 elderly patients including neurological, cardiovascular, pulmonary and geriatric patients [31] for a 6-month period. This difference could be justified by the different case mix in the two studies. The fall rate in orthopedic patients found in our study was just under 20%. This is in line with previously published papers. Chan and colleagues reported a fall rate of 17.2% after total knee replacement surgery [32] and Ikutomo and colleagues reported a fall rate of 36% after hip replacement surgery [33]. Neurological patients were more prone to experiencing a fall after discharge. For these patients, the equinus foot deformity, stiff knee and muscle overactivity are well known fall risk factors [34,35,36]. In our sample, nearly one out of three NR patients fell in the six months following their return home. Ng and colleagues reported a greater fall rate of approximately 50%. However, their study lasted 12 months, which was twice the time of our follow-up period.

A total of 5.6% of sample patients experienced a fracture subsequent to a fall, which is well in line with the fracture rate reported by Ng and colleagues (4%) [30]. Such results confirm the need for preventive actions to be undertaken by rehabilitation professionals such as: patient training prior to their return home [37]; fall risk assessment at discharge; educational interventions [31], and home safety assessment performed by PTs or OTs [38,39]. In line with this need, multifactorial fall prevention programs for people returning home after rehabilitation are currently being developed and tested [9,12,31].

There are limitations to this study. First, the patients’ fall history was collected by a survey at the 6-month mark. The assessment of the fall risk should have been repeated with each change in the patient’s condition, in the six months of follow-up, as recommended when monitoring patients’ risks. Worsening of health conditions, if any, were not detected in our dataset and this may have led to false negatives. Other relevant information is missing in our study, such as ongoing therapy, patients’ cognitive level and lifestyle. Moreover, collection of information on falls at six months after discharge could potentially underestimate the number of falls. A falls diary and monthly telephone calls, when doable, would be a more appropriate procedure [31]. Despite these limitations, the results of this study suggest that the use of the HIIFRM scale may be useful in spotting subjects at high risk of falling when discharging them to their home and who should receive some preventive intervention, linked to their positive risk factors. Moreover, patients can be informed about their fall risk status. Table 4 suggests that subjects may underestimate their condition and perform risky actions that may result in a fall.

Another limitation of this study is the limited sample size, which is however similar to that of many studies regarding this topic [40,41,42]. This was partially counteracted by the very high prevalence of the condition (a fall) in the sample. The preliminary estimate of sensitivity and specificity provided by this study allowed for the estimation of the sample size needed to conduct a comprehensive study of predictive ability of HIIFRM when administered to orthopedic patients at their discharge.

## 5. Conclusions

HIIFRM is a promising tool in identifying orthopedic patients at high risk of falling after discharge from orthopedic rehabilitation wards. In these patients, it could be used to plan immediate preventive interventions on their modifiable risk factors and to promote fall risk monitoring procedures. Since HIIFRM showed a very high specificity but a lower sensitivity, it could be used in association with functional fall risk assessment tools, as recommended in the conclusions published in recent systematic reviews.

## Figures and Tables

**Table 1:** Sample characteristics at discharge from rehabilitation wards.

Variable	Whole Sample Characteristics	Sample Characteristics Split by Wards		
		NR	OR	PR
Patients, *n*	85	26	46	13
Age, years, mean (SD); min, 25th, 50th, 75th percentile, max	67 (16); 20, 60, 72, 77, 91	56 (18); 20, 55, 64, 71, 91	70 (16); 20, 67, 74, 79, 91	69 (10); 54, 59, 72, 75, 86
Gender female/male; *n*	51/34	14/12	31/15	6/7
HIIFRM score; median (IQR); range	3 (4); 0–11	5 (4); 1–11	3 (4); 0–9	2 (1); 0–9
Subjects classified at high risk of falling; *n* (%)	35 (41%)	19 (73%)	14 (30%)	2 (15%)

Legend: NR, neurological rehabilitation; OR, orthopedic rehabilitation; PR, pulmonary rehabilitation.

**Table 2:** Occurrences of the HIIFRM (Hendrich Fall Risk Model II) items in subjects at discharge from NR (neurological rehabilitation), OR (orthopedic rehabilitation) and PR (pulmonary rehabilitation) wards. The “raise from chair” item is split for each score of the test.

Item	Score	Occurrences		
		NR (*n* = 26)	OR (*n* = 46)	PR (*n* = 13)
Confusion/Disorientation	(4)	19%	4%	8%
Symptomatic Depression	(2)	8%	20%	8%
Altered Elimination	(1)	35%	54%	46%
Dizziness/Vertigo	(1)	77%	41%	15%
Male Gender	(1)	46%	33%	54%
Antiepileptics	(2)	15%	7%	8%
Benzodiazepines	(1)	54%	35%	46%
Chair test = 0	(0)	23%	17%	69%
Chair test = 1	(1)	4%	7%	23%
Chair test = 3	(3)	15%	2%	8%
Chair test = 4	(4)	58%	26%	0%
Chair test = not feasible	(0)	0%	48%	0%

**Table 3:** Fallen subjects characteristics and falls consequences at the 6-month mark after discharge.

Variable	Whole Sample	Sample by Ward		
	(*n* = 85)	NR (*n* = 26)	OR (*n* = 46)	PR (*n* = 13)
Observed falls, *n* (%)	19 (22%)	9 (35%)	9 (20%)	1 (8%)
Age, years, mean (SD); min, 25th, 50th, 75th percentile, max	64 (18); 20, 56, 67, 79, 91	56 (19); 20, 54, 63, 67, 75	72 (15); 41, 73, 75, 79, 91	64
Fallen subjects median HIIFRM	6	5	7	9
Fractures, *n* (%fallen)	5 (26%)	3 (11%)	2 (4%)	-
Hospitalization, *n* (%fallen)	5 (26%)	3 (11%)	2 (4%)	-
Surgery, *n* (%fallen)	3 (17%)	2 (8%)	1 (2%)	-

**Table 4:** Description of fall causes and consequences.

Discharge Ward	HIIFRM Score	No. of Falls	Causes of Falls	Service Accessed	Fractures	Surgery
NR	11	1	Falling off the bed when adjusting position	none		
NR	9	4	#1 getting up from a chair; #2 getting up from a wheelchair; #3 getting up from a chair; #4 getting up from a wheelchair	GP		
NR	8	2	Slipping on a wet floor, trips when walking briskly and with the aid of a walking stick	ES	Leg	
NR	5	1	Epileptic seizure after getting up from a chair	ES; H	Leg	Yes
NR	5	1	Walking briskly with the aid of a walking stick and slipping on a wet floor	GP		
NR	5	2	#1 slips on a wet surface; #2 slips and falls in the bathroom	GP		
NR	3	1	Trips and falls down the stairs	ES; H	Femur	Yes
NR	2	1	Walking on a rough and uneven surface	GP		
NR	1	1	Walking on a wet floor	GP		
OR	9	2	#1 trips at home whilst walking without the aid of a walker; #2 trips outdoors whilst walking without the aid of a walker	GP		
OR	8	1	Hypotension caused by standing up to an upright position	GP		
OR	8	1	Epileptic seizure	ES; H	Traumatic Brain Injury	
OR	8	1	Getting dressed in the bathroom	ES; H	Femur	Yes
OR	7	1	Getting up and off the bed	GP		
OR	6	1	Rushing to the bathroom due to bowel incontinence	ES	Ribs	
OR	3	1	Fainting spell due additional medication intake (pain killers)	GP		
OR	3	1	Hopping on a bicycle and the skirt gets stuck in the gears or spokes	GP		
OR	2	1	Getting up from a lift chair or electric recliner	ES		
PR	9	1	Whilst walking	-		

Legend. ES, emergency service; H, hospitalization; GP, general practitioner; NR, neurological rehabilitation; OR, orthopedic rehabilitation; PR, pulmonary rehabilitation.

**Table 5:** Predictive ability of the HIIFRM administered at discharge from rehabilitation wards.

		Whole Sample			NR-Discharged Patients			OR-Discharged Patients			PR-Discharged Patients		
Predicted falls		True Falls			True Falls			True Falls			True Falls		
		Y	N	tot	Y	N	tot	Y	N	tot	Y	N	tot
	Y	13	22	35	6	13	19	6	8	14	1	1	2
	N	6	44	50	3	4	7	3	30	33	0	11	11
	tot	19	66	85	9	17	26	9	38	47	1	12	13
			C.I. 95%			C.I. 95%			C.I. 95%			C.I. 95%	
Se (%)		68%	43%	87%	60%	26%	88%	67%	30%	93%	-		
Sp (%)		67%	54%	78%	35%	15%	59%	79%	63%	90%	92%	65%	99%
PPV (%)		37%	27%	48%	32%	20%	45%	43%	26%	62%	-		
NPV (%)		88%	79%	95%	64%	40%	82%	91%	80%	96%	-		

Legend: Yes (Y), No (N), total (tot), sensitivity (Se), specificity (Sp), positive and negative predicted values (PPV, NPV).

## Data Availability

The data presented in this study are available on request from the corresponding author.
